# Prepandemic Physical Activity and Risk of COVID-19 Diagnosis and Hospitalization in Older Adults

**DOI:** 10.1001/jamanetworkopen.2023.55808

**Published:** 2024-02-13

**Authors:** Dennis Muñoz-Vergara, Peter M. Wayne, Eunjung Kim, I-Min Lee, Julie E. Buring, JoAnn E. Manson, Howard D. Sesso

**Affiliations:** 1 Osher Center for Integrative Medicine, Brigham and Women’s Hospital, Harvard Medical School, Boston, Massachusetts; 2 Division of Preventive Medicine, Brigham and Women’s Hospital, Harvard Medical School, Boston, Massachusetts; 3 Department of Epidemiology, Harvard T.H. Chan School of Public Health, Boston, Massachusetts

## Abstract

**Importance:**

Higher prepandemic physical activity (PA) levels have been associated with lower risk and severity of COVID-19.

**Objective:**

To investigate the association between self-reported prepandemic PA levels and the risk and severity of COVID-19 in older US adults.

**Design, Setting, and Participants:**

This cohort study combined cohorts from 3 ongoing prospective randomized clinical trials of US adults aged 45 years or older who provided prepandemic self-reports of baseline leisure-time PA and risk factors for COVID-19 outcomes using the most recent questionnaire completed as of December 31, 2019, as the baseline PA assessment. In multiple surveys from May 2020 through May 2022, participants indicated whether they had at least 1 positive COVID-19 test result or were diagnosed with or hospitalized for COVID-19.

**Exposure:**

Prepandemic PA, categorized into 3 groups by metabolic equivalent hours per week: inactive (0-3.5), insufficiently active (>3.5 to <7.5), and sufficiently active (≥7.5).

**Main Outcome and Measures:**

Primary outcomes were risk of COVID-19 and hospitalization for COVID-19. Multivariable logistic regression was used to estimate odd ratios (ORs) and 95% CIs for the association of COVID-19 diagnosis and/or hospitalization with each of the 2 upper PA categories vs the lowest PA category.

**Results:**

The pooled cohort included 61 557 participants (mean [SD] age, 75.7 [6.4] years; 70.7% female), 20.2% of whom were inactive; 11.4%, insufficiently active; and 68.5%, sufficiently active. A total of 5890 confirmed incident cases of COVID-19 were reported through May 2022, including 626 hospitalizations. After controlling for demographics, body mass index, lifestyle factors, comorbidities, and medications used, compared with inactive individuals, those insufficiently active had no significant reduction in infection (OR, 0.96; 95% CI, 0.86-1.06) or hospitalization (OR, 0.98; 95% CI, 0.76-1.28), whereas those sufficiently active had a significant reduction in infection (OR, 0.90; 95% CI, 0.84-0.97) and hospitalization (OR, 0.73; 95% CI, 0.60-0.90). In subgroup analyses, the association between PA and SARS-CoV-2 infection differed by sex, with only sufficiently active women having decreased odds (OR, 0.87; 95% CI, 0.79-0.95; *P* = .04 for interaction).

**Conclusions and Relevance:**

In this cohort study of adults aged 45 years or older, those who adhered to PA guidelines before the pandemic had lower odds of developing or being hospitalized for COVID-19. Thus, higher prepandemic PA levels may be associated with reduced odds of SARS-CoV-2 infection and hospitalization for COVID-19.

## Key Points

**Question:**

Are higher prepandemic physical activity (PA) levels associated with lower risk of developing or being hospitalized for COVID-19?

**Findings:**

In this cohort study of 61 557 women and men aged 45 years or older who reported 5890 incident cases of COVID-19 and 626 hospitalizations, those who achieved at least 7.5 metabolic equivalent hours per week of PA before the pandemic had significantly reduced odds of COVID-19 diagnosis and hospitalization compared with the inactive group.

**Meaning:**

Higher prepandemic PA levels were associated with lower odds of developing and being hospitalized for COVID-19.

## Teaser

This cohort study uses pooled data from 3 randomized clinical trials to assess the association between physical activity levels before the COVID-19 pandemic and subsequent odds of COVID-19 diagnosis and hospitalization.

## Introduction

Research supports physical activity (PA) for health and reductions in major morbidity and mortality.^1,2^ Adherence to US guidelines of at least 150 min/wk of moderate to vigorous PA may help prevent or mitigate effects of cardiovascular disease (CVD), cancer, type 2 diabetes, and other chronic conditions.^3,4,5,6^ Some health benefits of PA are attributable to the delay of age-related immunosenescence, reduced low-grade systemic inflammation, and boosted immunity.^7,8,9,10^ However, significant gaps exist in the evidence that PA protects against infectious diseases.

The COVID-19 pandemic provides a unique opportunity to address the association of PA with infection.^8,11,12,13,14^ A recent study found that meeting PA guidelines before COVID-19 diagnosis was associated with fewer severe outcomes, including hospitalization, admission to intensive care, and death.^15^ Other studies have assessed potential for PA and other healthy lifestyle factors, such as healthy body weight, limited alcohol intake, or high-quality diet, to synergically boost the immune system to prevent or ameliorate the severity of COVID-19.^16,17,18^ However, the generalizability of these findings to older adults is limited in studies published to date.

This study pooled data from 3 large, ongoing prospective trial cohorts of older adults who self-reported PA levels before the COVID-19 pandemic and were prospectively followed up for SARS-CoV-2 infection and hospitalization through 2021. We hypothesized that higher levels of prepandemic PA would be associated with reduced risk of SARS-CoV-2 infection and COVID-19 hospitalization.

## Methods

### Study Design

This prospective cohort study combined participants from 3 large-scale randomized clinical trials (RCTs) directed by our research group: the Cocoa Supplement and Multivitamin Outcomes Study (COSMOS), a double-blind, placebo-controlled, factorial RCT of a cocoa extract and multivitamin supplement in the prevention of CVD and cancer among 21 442 women aged 65 years or older and men aged 60 or older^19,20^; the Vitamin D and Omega-3 Trial (VITAL), a double-blind, placebo-controlled, factorial RCT of fatty acid supplements in the prevention of CVD and cancer among 25 871 women aged 55 or older and men aged 50 or older^21^; and the Women’s Health Study (WHS), a double-blind, placebo-controlled, factorial RCT of low-dose aspirin and vitamin E in the primary prevention of CVD and cancer among 39 876 US female health professionals aged 45 or older.^22,23,24^ The institutional review board at Mass General Brigham approved all study-related activities. Written informed consent was obtained from participants in all 3 trials. We followed the Strengthening the Reporting of Observational Studies in Epidemiology (STROBE) reporting guideline.^25^

### Cohort With COVID-19

In May, June, and August 2020, we sent 3 separate online REDCap surveys asking about COVID-19 symptoms, testing, diagnoses, treatments, severity of illness, and risk factors to COSMOS, VITAL, and WHS participants who provided email addresses and were willing to be contacted by email to complete surveys online (eFigure in Supplement 1). Those who responded to at least 1 survey or study follow-up questionnaire and had PA measurements were included. In addition, we repeated questions on COVID-19 testing, diagnoses, and hospitalization from the previous year on regular annual follow-up questionnaires for each cohort in 2021 and 2022.

### Assessment of Prepandemic PA

To assess prepandemic self-reported PA (as validated in previous studies^3,19,22^) and other relevant risk factors, we leveraged long-term prospective questionnaire data in COSMOS, VITAL, and WHS and used the most recent annual questionnaire on or before December 31, 2019 (median year of completion, 2017; range, 2003-2019), as the baseline for the PA assessment. We asked 2 questions about typical PA habits over the past year: (1) “During the past year, what was your approximate average time (in minutes) per week spent at each of the following recreational activities (eg, walking, jogging, running, aerobic exercise, etc)?” and (2) “On average, how many flights of stairs (1 flight is typically 10 steps) do you climb daily?” Participant responses were converted into total reported PA as metabolic equivalent [MET] hours per week by assigning MET values to different activities and estimating total energy expenditure based on reported duration and frequency of those activities for a given week.^3,4,5,6^ Based on US and World Health Organization (WHO) PA guidelines,^4,6^ we created 3 PA categories, in MET-h/wk, for our analyses: inactive (0-3.5), insufficiently active (>3.5 to <7.5), and sufficiently active (≥7.5). Our determination for being at least sufficiently active was based on the lower bounds for recommended moderate to vigorous PA (≥3 METs for 150 minutes per week), resulting in 450 MET-min/wk, or 7.5 MET-h/wk.^6^

### Other Covariates

Other self-reported covariates included those collected at the most recent annual questionnaire closest to December 31, 2019, before the start of the COVID-19 pandemic. Demographic variables included sex; age; and race and ethnicity (African American or Black; Asian or Native American; Hispanic or Latinx; non-Hispanic White; and other [ie, different from the 4 other categories], unknown, or not reported), ascertained by self-report and included to explore racial and ethnic differences in subgroup analyses. We also examined educational attainment (no high school, high school, some college, college graduate, and postcollege) and income (<$30 000, $30 000 to <$50 000, $50 000 to <$100 000, and ≥$100 000).^19,21,22^ Lifestyle factors included smoking status (never, past, and current) and alcohol consumption (rarely or never, monthly, weekly, and daily). Body mass index (BMI, calculated as weight in kilograms divided by height in meters squared) was based on self-reported height and weight. We also considered several comorbidities and medications used at the end of 2019. Comorbidities were adjudicated by a committee of physicians and investigators according to standardized procedures (cancer, myocardial infarction, and stroke)^26^ or based on self-reports of medications and/or diagnoses (history of diabetes, hypertension, or statin use). Current medications used included nonsteroidal anti-inflammatory drugs and aspirin.

### Risk and Severity of COVID-19

Based on 3 REDCap surveys in 2020 and annual follow-up questionnaires, we classified participants as having had COVID-19 if they reported a test positive for SARS-CoV-2 or its antibodies, were told by a health care professional that they were probably or definitely diagnosed with COVID-19, or reported being hospitalized for COVID-19 on any of the questionnaires. Participants also provided the month and year of a positive test result, diagnosis, and/or hospitalization for COVID-19. We used the date of questionnaire return for missing dates. We separately defined severity of COVID-19 based on whether individuals reported COVID-19 hospitalization. We used information on all reported diagnoses, test results, and hospitalizations for COVID-19 through May 10, 2022.

### Statistical Analysis

Our primary outcomes were risk of SARS-CoV-2 infection and hospitalization due to COVID-19. We compared demographics, lifestyle factors, comorbidities, and medications among the 3 PA groups using analysis of variance tests for continuous variables and χ^2^ tests for categorical variables. Multivariable logistic regression models estimated the odd ratios (ORs) and 95% CIs for the association of each of the 2 upper PA categories (vs the lowest PA category) with SARS-CoV-2 infection and hospitalization for COVID-19 (model 1 adjusted for demographic characteristics, model 2 added lifestyle factors, and model 3 added comorbidities and medications). We also considered a priori subgroup analyses by sex, BMI, race and ethnicity, and income^27^ and post hoc analyses by history of CVD and cancer. We evaluated these potential multiplicative modifications of associations using the Wald test for homogeneity. Two-sided *P* < .05 was considered significant.

We conducted 3 sensitivity analyses to evaluate the stability and reliability of our results. First, we restricted analyses through December 31, 2020, before SARS-CoV-2 vaccines became widely available in the US. Second, we conducted analyses adding SARS-CoV-2 vaccination status, initially collected on annual questionnaires to all participants starting January 2021. Third, we combined the consistently inactive and insufficiently active groups vs the sufficiently active group. Analyses were performed using SAS, version 9.4 (SAS Institute Inc).

## Results

A total of 69 604 adults aged 45 years or older were invited to participate, of whom 61 557 (88.4%) responded and comprised the cohort for our analyses. eTable 1 in Supplement 1 provides the number of individuals who reported positive test results and/or hospitalizations per study, along with other demographic characteristics. As of December 31, 2019, the cohort had a mean (SD) age of 75.7 (6.4) years; 70.7% were female, and 29.3% were male (Table 1). For PA, 20.2% of participants reported being inactive; 11.4%, insufficiently active; and 68.5%, sufficiently active. A total of 7.5% of participants were African American or Black; 2.1%, Asian or Native American; 2.3%, Hispanic or Latinx; 87.2%, non-Hispanic White; and 0.9%, other, unknown, or unreported race and ethnicity. Also, 24.1% of the cohort reported a BMI of 30 or greater. Participants with higher educational and income levels and those who never smoked were more likely to report sufficient PA. During follow-up, there were 5890 incident cases of COVID-19 and 626 of hospitalization due to COVID-19 (eFigure in Supplement 1).

**Table 1.** Participant Characteristics as of December 31, 2019, by Physical Activity Levels Measured in MET Hours per Week

Characteristic	Participants^a^			
	All (N = 61 557)	Inactive (n = 12 405)	Insufficiently active (n = 6993)	Sufficiently active (n = 42 159)
Sex				
Men	18 047 (29.3)	2756 (22.2)	1874 (26.8)	13 417 (31.8)
Women	43 510 (70.7)	9649 (77.8)	5119 (73.2)	28 742 (68.2)
Age, mean (SD), y	75.7 (6.4)	76.9 (6.8)	76.3 (6.6)	75.3 (6.2)
Race and ethnicity				
African American or Black	4638 (7.5)	1359 (11.0)	640 (9.2)	2639 (6.3)
Asian or Native American	1269 (2.1)	205 (1.7)	156 (2.2)	908 (2.2)
Hispanic or Latinx	1415 (2.3)	319 (2.6)	184 (2.6)	912 (2.2)
Non-Hispanic White	53 670 (87.2)	10 396 (83.8)	5945 (85.0)	37 329 (88.5)
Other, unknown, or not reported^b^	565 (0.9)	126 (1.0)	68 (0.9)	371 (1.0)
BMI				
<30	46 174 (75.0)	7325 (59.0)	4790 (68.5)	34 059 (80.8)
≥30	14 829 (24.1)	4951 (40.0)	2140 (30.6)	7738 (18.4)
Missing	554 (0.9)	129 (1.0)	63 (0.9)	362 (0.9)
Educational level				
High school	4000 (6.6)	1470 (11.9)	559 (8.0)	1971 (4.7)
Some college	10 747 (17.5)	2990 (24.1)	1427 (20.4)	6330 (15.0)
College graduate	21 879 (35.5)	4566 (36.8)	2595 (37.1)	14 718 (34.9)
Postcollege	24 357 (39.6)	3246 (26.2)	2341 (33.5)	18 770 (44.5)
Missing	574 (0.9)	133 (1.1)	71 (1.0)	370 (0.9)
Annual income, $				
<30 000	6699 (10.9)	2309 (18.6)	924 (13.2)	3466 (8.2)
30 000 to <50 000	11 300 (18.4)	2858 (23.0)	1527 (21.8)	6915 (16.4)
50 000 to <100 000	26 573 (43.2)	4733 (38.2)	2917 (41.7)	18 923 (44.9)
≥100 000	11 252 (18.3)	1211 (9.8)	932 (13.3)	9109 (21.6)
Missing	6733 (10.9)	1294 (10.4)	693 (10.0)	3746 (8.9)
Smoking status				
Never	32 176 (52.3)	6110 (49.3)	3580 (51.2)	22 486 (53.3)
Past	26 961 (43.8)	5510 (44.4)	3088 (44.2)	18 363 (43.6)
Current	1912 (3.1)	689 (5.6)	261 (3.7)	962 (2.3)
Missing	508 (0.8)	96 (0.8)	64 (0.9)	348 (0.8)
Alcohol use				
Rarely or never	20 612 (33.5)	5785 (46.6)	2743 (39.2)	12 084 (28.7)
Monthly	5092 (8.3)	1131 (9.1)	612 (8.8)	3349 (7.9)
Weekly	21 915 (35.6)	3540 (28.5)	2360 (33.7)	16 015 (38.0)
Daily	13 554 (22.0)	1853 (14.9)	1222 (17.5)	10 479 (24.9)
Missing	384 (0.6)	96 (0.8)	56 (0.8)	232 (0.6)
Comorbidities				
Diabetes	9306 (15.1)	3050 (24.6)	1292 (18.5)	4964 (11.8)
Hypertension	41 723 (67.8)	9939 (80.1)	5243 (75.0)	26 541 (63.0)
Malignant cancer^c^	4639 (7.5)	1121 (9.0)	536 (7.7)	2982 (7.1)
Myocardial infarction	707 (1.2)	197 (1.6)	106 (1.5)	404 (1.0)
Stroke	662 (1.1)	203 (1.6)	79 (1.1)	380 (0.9)
Medications used				
NSAIDs	20 210 (32.8)	4077 (32.9)	2330 (33.3)	13 803 (32.7)
Aspirin	24 146 (39.2)	5260 (42.4)	2877 (41.1)	16 009 (38.0)
Statins	27 190 (44.2)	5889 (47.5)	3249 (46.5)	18 052 (42.8)

Abbreviations: BMI, body mass index (calculated as weight in kilograms divided by height in meters squared); MET, metabolic equivalent; NSAID, nonsteroidal anti-inflammatory drug.

^a^ Data are presented as number (percentage) of participants unless otherwise indicated. Percentages may not sum to 100 because of rounding. Inactive was 0 to 3.5 MET-h/wk; insufficiently active, more than 3.5 to less than 7.5 MET-h/wk; and sufficiently active, 7.5 or more MET-h/wk.

^b^ Racial and ethnic groups different from the 4 categories listed.

^c^ Except nonmelanoma skin cancer.

In all models, sufficiently active participants had significantly lower odds of SARS-CoV-2 infection (eg, model 3: OR, 0.90; 95% CI, 0.84-0.97; *P* = .01) compared with those who were inactive (Table 2). In the insufficiently active group, PA was not associated with odds of SARS-CoV-2 infection compared with the inactive group in any model (eg, model 3: OR, 0.96; 95% CI, 0.86-1.06; *P* = .89). Participants who were sufficiently active had consistently lower odds of hospitalization due to COVID-19 (eg, model 3: OR, 0.73; 95% CI, 0.60-0.90; *P* = .001) compared with those who were inactive (Table 3). In the insufficiently active group, there was no association of PA with odds of COVID-19 hospitalization (eg, model 3: OR, 0.98; 95% CI, 0.76-1.28; *P* = .25).

**Table 2.** Adjusted Odds of COVID-19 by Level of Physical Activity Before the COVID-19 Pandemic

Physical activity category^a^	Participants, No.	COVID-19 events, No.	Adjusted OR (95% CI)		
			Model 1^b^	Model 2^c^	Model 3^d^
Inactive	12 405	1293	1 [Reference]	1 [Reference]	1 [Reference]
Insufficiently active	6993	699	0.97 (0.88-1.07)	0.96 (0.87-1.06)	0.96 (0.86-1.06)
Sufficiently active	42 159	3898	0.90 (0.84-0.97)	0.90 (0.84-0.97)	0.90 (0.84-0.97)

Abbreviations: MET, metabolic equivalent; OR, odds ratio.

^a^ Inactive was 0 to 3.5 MET-h/wk; insufficiently active, more than 3.5 to less than 7.5 MET-h/wk; and sufficiently active, 7.5 or more MET-h/wk.

^b^ Adjusted for demographic characteristics (sex, age, race and ethnicity, educational level, and income) and body mass index.

^c^ Adjusted for demographic characteristics, body mass index, and lifestyle factors (smoking status, alcohol intake).

^d^ Fully adjusted for demographic characteristics, body mass index, lifestyle factors (smoking status, alcohol intake), comorbidities (history of diabetes, hypertension, malignant cancer, myocardial infarction, or stroke), and medication use (nonsteroidal anti-inflammatory drugs, aspirin, and statins).

**Table 3.** Adjusted Odds of Hospitalization Due to COVID-19 by Level of Physical Activity Before the COVID-19 Pandemic

Physical activity category^a^	Participants, No.	COVID-19 hospitalizations, No.	Adjusted OR (95% CI)		
			Model 1^b^	Model 2^c^	Model 3^d^
Inactive	12 405	203	1 [Reference]	1 [Reference]	1 [Reference]
Insufficiently active	6993	91	0.95 (0.73-1.22)	0.98 (0.76-1.27)	0.98 (0.76-1.28)
Sufficiently active	42 159	332	0.71 (0.59-0.87)	0.74 (0.61-0.90)	0.73 (0.60-0.90)

Abbreviations: MET, metabolic equivalent; OR, odds ratio.

^a^ Inactive was 0 to 3.5 MET-h/wk; insufficiently active, more than 3.5 to less than 7.5 MET-h/wk; and sufficiently active, 7.5 or more MET-h/wk.

^b^ Adjusted for demographic characteristics (sex, age, race and ethnicity, educational level, and income) and body mass index.

^c^ Adjusted for demographic characteristics, body mass index, and lifestyle factors (smoking status, alcohol intake).

^d^ Fully adjusted for demographic characteristics, body mass index, lifestyle factors (smoking status, alcohol intake), comorbidities (history of diabetes, hypertension, malignant cancer, myocardial infarction, or stroke), and medication use (nonsteroidal anti-inflammatory drugs, aspirin, and statins).

In subgroup analyses, the association between PA and COVID-19 differed by sex. Women who were sufficiently active had decreased odds of SARS-CoV-2 infection compared with inactive women (OR, 0.87; 95% CI, 0.79-0.95; *P* = .04 for interaction) (Table 4). There was no association in men. No evidence of association modification was observed for BMI, race and ethnicity, income, and CVD or cancer. In sensitivity analyses restricting results to follow-up through December 2020 (before widespread vaccination programs), the number of cases was reduced by 53.5% (2739 cases). There was no association between sufficient PA and SARS-CoV-2 infection in any model (eg, model 3: OR, 0.98; 95% CI, 0.88-1.09; *P* = .58). The lack of an association persisted for the insufficiently active group (eg, model 3: OR, 1.01; 95% CI, 0.88-1.17; *P* = .72). However, the odds of COVID-19 hospitalization through December 2020 were significantly lower for the sufficiently active than for the inactive group (eg, model 3: OR, 0.64; 95% CI, 0.49-0.83; *P* = .003) (eTables 2 and 3 in Supplement 1).

**Table 4.** Modification of the Association of Physical Activity With Risk of COVID-19 by Selected Covariates^a^

Covariate	Inactive group (n = 12 405)^b^	Insufficiently active group (n = 6993)		*P* value	Sufficiently active group (n = 42 159)		*P* value for interaction^c^
	With COVID-19, No./total No. (%)	With COVID-19, No./total No. (%)	Adjusted OR (95% CI)		With COVID-19, No./total No. (%)	Adjusted OR (95% CI)	
Sex							
Men	343/2756 (12.4)	244/1874 (13.0)	1.07 (0.89-1.29)	.37	1563/13 417 (11.6)	0.99 (0.86-1.14)	.04
Women	950/9649 (9.8)	455/5119 (8.9)	0.91 (0.81-1.03)	.68	2335/28 742 (8.1)	0.87 (0.79-0.95)	
BMI							
<30	711/7325 (9.7)	423/4790 (8.8)	0.86 (0.76-0.98)	.28	2981/34 059 (8.8)	0.84 (0.77-0.92)	.09
≥30	568/4951 (11.5)	269/2140 (12.6)	1.10 (0.94-1.30)	.12	873/7738 (11.3)	0.96 (0.85-1.09)	
Race and ethnicity							
African American or Black	194/1359 (14.3)	90/640 (14.1)	0.93 (0.69-1.27)	.92	323/2639 (12.2)	0.85 (0.68-1.06)	.87
Non-Hispanic White	1028/10 396 (9.9)	560/5945 (9.4)	0.95 (0.85-1.07)	.99	3364/37 329 (9.0)	0.90 (0.83-0.98)	
Other^d^	71/650 (10.9)	49/408 (12.0)	1.09 (0.71-1.69)	.60	211/2191 (9.0)	0.98 (0.70-1.37)	
Annual income, $							
<100 000	1064/9900 (10.7)	553/5368 (10.3)	0.94 (0.84-1.05)	<.99	2723/29 304 (9.3)	0.88 (0.81-0.95)	.12
≥100 000	107/1211 (8.8)	88/932 (9.4)	1.12 (0.82-1.53)	.55	848/9109 (9.3)	1.08 (0.85-1.36)	
CVD or cancer							
No	1154/10 958 (10.5)	647/6304 (10.3)	0.97 (0.87-1.08)	.62	3589/38 492 (9.3)	0.90 (0.83-0.97)	.52
Yes	139/1447 (9.6)	52/689 (7.5)	0.84 (0.60-1.19)	.33	309/3667 (8.4)	0.96 (0.76-1.22)	

Abbreviations: BMI, body mass index (calculated as weight in kilograms divided by height in meters squared); CVD, cardiovascular disease; MET, metabolic equivalent; OR, odds ratio.

^a^ Inactive was 0 to 3.5 MET-h/wk; insufficiently active, more than 3.5 to less than 7.5 MET-h/wk; and sufficiently active, 7.5 or more MET-h/wk.

^b^ Reference group.

^c^ Interaction terms were explored using model 3, with ORs adjusted for demographics (sex, age, race and ethnicity, educational level, and income), BMI, lifestyle factors (smoking status, alcohol intake), comorbidities (history of diabetes, hypertension, malignant cancer, myocardial infarction, or stroke), and medication use (nonsteroidal anti-inflammatory drugs, aspirin, and statins).

^d^ Included Asian or Native American, Hispanic or Latinx, or any other race and ethnicity as well as unknown or unreported race and ethnicity.

We also conducted a sensitivity analysis adjusting for potential confounding by SARS-CoV-2 vaccination status in model 3. The ORs for infection and hospitalization did not substantially change for the sufficiently active group (eg, infection: OR, 0.89; 95% CI, 0.82-0.96; *P* = .007; hospitalization: OR, 0.74; 95% CI, 0.60-0.92; *P* = .005) (eTables 4 and 5 in Supplement 1). SARS-CoV-2 vaccination was associated with substantially decreased odds of infection (OR, 0.55; 95% CI, 0.50-0.61; *P* < .001) and hospitalization (OR, 0.37; 95% CI, 0.30-0.47; *P* < .001) for all PA levels assessed before the COVID-19 pandemic. Our third sensitivity analysis combined the consistently inactive and insufficiently active groups to assess whether sufficient PA was still associated with lower odds of SARS-CoV-2 infection and COVID-19 hospitalization in model 3. The decreased ORs for infection and hospitalization did not substantially change for the sufficiently active group (eg, infection: OR, 0.92; 95% CI, 0.86-0.98, *P* = .01; hospitalization: OR, 0.74; 95% CI, 0.62-0.89; *P* = .001) (eTables 6 and 7 in Supplement 1).

## Discussion

In this cohort study, sufficiently active participants had significantly reduced odds of SARS-CoV-2 infection and of hospitalization due to COVID-19 compared with those who were inactive. This difference was not observed between the insufficiently active and inactive groups. Results were robust across models adjusting for multiple covariates. These findings parallel a previously reported association between high PA levels and reduced odds of infection and mortality due to viral and bacterial pneumonia.^28^ Other studies have reported potential inverse associations between PA levels and risk and/or severity of COVID-19; their results included a wider age range in adult populations.^15,16,17,18,29^ Our findings extend the understanding of the association between PA and vulnerability to infections, specifically with highly infectious respiratory viruses, among older adults.^10^

We found reduced odds of infection across all 3 models when comparing the sufficiently active vs inactive groups. There was no apparent benefit of PA in the insufficiently active group. This suggests that the association between PA and COVID-19 may depend on the amount, intensity, and/or type of PA.^11,15^ Cho et al^30^ found equivalent ORs for COVID-19 among individuals engaging in both moderate (10 MET-h/wk) and vigorous (17.5 MET-h/wk) PA. Ahmadi et al^31^ reported inverse associations between PA and COVID-19 for both insufficiently (<10 MET-h/wk) and sufficiently (≥10 MET-h/wk) active individuals. Rowlands et al^32^ also described higher odds of nonsevere COVID-19 when adjusting for PA intensity (accelerometer-assessed) and self-reported PA levels (moderate to vigorous PA [MVPA]: 7.5-15 MET-h/wk). Lee et al^33^ reported a consistent inverse association of PA with risk of COVID-19 among individuals practicing both aerobic and muscle strengthening exercises but not either alone and among those fulfilling the recommended range of 8.3 to 17 MET-h/wk. Regardless of differences in study designs, population characteristics, and PA or risk of SARS-CoV-2 infection and severity assessments, these studies are consistent with our findings regarding the inverse association between PA levels and risk of SARS-CoV-2 infection and COVID-19 severity. Also, our findings parallel the conclusions from 2 systematic reviews and meta-analyses characterizing the association between PA and risk of infection with other community-acquired respiratory infectious viruses, such as influenza and current variants of SARS-CoV-2.^10,13^

For the association between PA and hospitalization due to COVID-19, our results align with those reported by English and Scottish health surveys, which also found an inverse association between MVPA (≥150-minute/wk) and COVID-19 mortality among 97 844 participants who, on average, were 56 years of age.^34^ A study of comparatively younger adults also found an association between PA and hospitalization due to COVID-19 among those consistently meeting PA guidelines, even after multivariable adjustment.^15^ A study characterizing associations between accelerometer-assessed PA and severe COVID-19 cases (ie, hospitalization or death) found lower odds of severe cases when adjusting for intensity and MVPA (7.5-15 MET-h/wk).^32^ Other studies reported similar patterns.^18,30,33,35^ Therefore, PA may prevent more severe cases of COVID-19 among those at greater risk of major morbidity and mortality, potentially explaining the lower odds of COVID-19 hospitalization among those meeting PA guidelines.^31^

Our subgroup analyses suggest that the inverse association between PA and COVID-19 outcomes may be greater among women, potentially due to differences in respiratory system physiology.^36^ As other studies indicated that older age, male sex, ethnicity status, low socioeconomic status, and having multiple morbidities were associated with higher risk of COVID-19 and more severe cases,^31,32,33,37,38,39,40^ future studies should clarify whether the role of PA extends to both short- and long-term COVID-19–related outcomes in these groups.

When we restricted our follow-up through December 2020 to examine risk of infection before the introduction of COVID-19 vaccines, the inverse associations of prepandemic PA with COVID-19 were absent, possibly suggesting residual confounding; for example, behavioral and psychosocial factors may have modified the association between PA and risk of SARS-CoV-2 infection.^41^ When we added vaccination status to model 3, the inverse association between meeting PA guidelines and COVID-19 was sustained. A previous study reported similar findings in a younger population.^16^ Moreover, in agreement with previous studies,^29,42,43^ the association of the COVID-19 vaccine with reduced risk of infection and hospitalization were evident regardless of PA status. Likewise, when we compared the combined inactive and insufficiently active groups with the sufficiently active group, the inverse associations remained. Previous studies also indicated the relevance of PA parameters in meeting PA guidelines (eg, ≥7.5 MET-h/wk).^4,32^

Increased PA may protect against COVID-19 and other infectious diseases through various mechanisms. It enhances immune surveillance mechanisms by increasing the activity of natural killer cells and neutrophils and the number of circulating monocytes and lymphocytes and by modulating inflammatory processes through different mediators, such as cytokines, myokines, immunoglobulins (Igs), cortisol, and oxylipins.^7,8,9,31,33^ Moreover, the increment of blood flow during PA biomechanically augments the lamina shear stress over endothelial cells, which triggers the production and bioavailability of nitric oxide, counteracting the oxidative and proinflammatory effect of SARS-CoV-2 infection.^44,45^ Physical activity also improves neurocognitive functioning and well-being, slows neurodegeneration, and optimizes stress response.^46^ In the myofascial system, the immunological and neurological pathways interplay by promoting muscle-derived anti-inflammatory interleukin 6 (ie, myokine production), long-term reduction of proinflammatory mediators, and release of endocannabinoids.^46,47,48^ Physical activity increases levels of salivary IgA, an antibody known to protect against respiratory viruses.^10,49,50^ Still, the exact mechanisms by which PA and more specific components of PA (eg, frequency, duration, intensity, and type) affect these physiological pathways warrant further investigation.^8,11,12,14^

### Strengths and Limitations

This study has strengths. The prospective design allowed us to define PA using validated questionnaires before the COVID-19 pandemic for the subsequent risk of COVID-19 and hospitalization due to COVID-19 as collected during the pandemic via multiple longitudinal surveys. Furthermore, we adjusted for a range of demographic, lifestyle, and clinical factors defined just before the COVID-19 pandemic.

Several limitations should be considered. First, the comparatively higher prevalence of sufficiently active participants in the combined cohort may reflect inherent volunteer bias for initially healthier individuals originally recruited and randomized into long-term clinical trials with continued follow-up.^19,22,51^ Second, because PA levels were self-reported, information inaccuracies (eg, random misclassification) could have penalized the insufficiently active group.^2^ Third, we likely underestimated COVID-19 cases due to missed asymptomatic cases without available serologic data and to underreporting typically seen in prospective studies. Longer follow-up for COVID-19 outcomes would have increased case counts, but the wider-spread integration of vaccines would have made it more difficult to isolate the role of PA. Furthermore, we did not account for changes in PA before and during the pandemic. Fourth, our definition of COVID-19 severity relied on hospitalization alone; however, some individuals with moderate or severe cases may not have been hospitalized. Fifth, we could not rule out the possibility of unmeasured confounders associated with high PA, including wearing masks, social distancing, and other protective behaviors,^52,53,54^ despite extensive control for known confounders.^55^ Sixth, although some participants were enrolled in more than 1 of the included studies, our main findings for risk of COVID-19 and hospitalization were unchanged in sensitivity analyses limited to those in 1 study only. Seventh, the cohort corresponded to a subset of participants from 3 parent RCTs and was largely a non-Hispanic White population (ie, sampling bias) recruited for specific purposes (eg, the WHS recruited only females); the generalizability of our findings to groups with different genders, ages, races and ethnicities, and comorbidities (ie, healthy volunteer bias) warrants further study.^56^

## Conclusions

In this cohort study of adults aged 45 years or older, meeting PA guidelines was associated with significantly lower odds of developing and being hospitalized for COVID-19. Future studies including quantitative control of PA parameters, broader racial and ethnic diversity, and information from other potential confounders (eg, sleep quality, dietary patterns, access to health care, and preventive behaviors) are warranted.

## Notes

Supplement 1. **eTable 1.** Characteristics of Preventive Medicine Trial Cohort Participants and Reported COVID-19 Cases to Date **eTable 2.** Adjusted Odds of COVID-19 by Level of Physical Activity Before the COVID-19 Pandemic, Restricting Follow-Up Through December 2020 **eTable 3.** Adjusted Odds of COVID-19 Hospitalization by Level of Physical Activity Before the COVID-19 Pandemic, Restricting Follow-Up Through December 2020 **eTable 4.** Adjusted Odds of COVID-19 by Level of Physical Activity Before the COVID-19 Pandemic, With Additional Adjustment for SARS-CoV-2 Vaccination Status **eTable 5.** Adjusted Odds of COVID-19 Hospitalization by Level of Physical Activity Before the COVID-19 Pandemic, With Additional Adjustment for SARS-CoV-2 Vaccination Status **eTable 6.** Adjusted Odds of COVID-19 by Level of Physical Activity Before the COVID-19 Pandemic, Combining Consistently Inactive and Insufficiently Active Participants in 1 Group vs Sufficiently Active **eTable 7.** Adjusted Odds of COVID-19 Hospitalization by Level of Physical Activity Before the COVID-19 Pandemic, Combining Consistently Inactive and Insufficiently Active Participants in 1 Group vs Sufficiently Active **eFigure.** Data Collection and Completeness of COVID-19 Outcomes

Supplement 2.
Data Sharing Statement
