# Supplementary material for: Prepandemic Physical Activity and Risk of COVID-19 Diagnosis and Hospitalization in Older Adults
Source: JAMA Netw Open. 2024 Feb 13;7(2):e2355808. doi: 10.1001/jamanetworkopen.2023.55808 (PMC10865155; doi:10.1001/jamanetworkopen.2023.55808)
Supplement: Supplement 1. — eTable 1. Characteristics of Preventive Medicine Trial Cohort Participants and Reported COVID-19 Cases to Date eTable 2. Adjusted Odds of COVID-19 by Level of Physical Activity Before the COVID-19 Pandemic, Restricting Follow-Up Through December 2020 eTable 3. Adjusted Odds of COVID-19 Hospitalization by Level of Physical Activity Before the COVID-19 Pandemic, Restricting Follow-Up Through December 2020 eTable 4. Adjusted Odds of COVID-19 by Level of Physical Activity Before the COVID-19 Pandemic, With Additional Adjustment for SARS-CoV-2 Vaccination Status eTable 5. Adjusted Odds of COVID-19 Hospitalization by Level of Physical Activity Before the COVID-19 Pandemic, With Additional Adjustment for SARS-CoV-2 Vaccination Status eTable 6. Adjusted Odds of COVID-19 by Level of Physical Activity Before the COVID-19 Pandemic, Combining Consistently Inactive and Insufficiently Active Participants in 1 Group vs Sufficiently Active eTable 7. Adjusted Odds of COVID-19 Hospitalization by Level of Physical Activity Before the COVID-19 Pandemic, Combining Consistently Inactive and Insufficiently Active Participants in 1 Group vs Sufficiently Active eFigure. Data Collection and Completeness of COVID-19 Outcomes [file jamanetwopen-e2355808-s001.pdf]

Supplementary Online Content

Muñoz-Vergara D, Wayne PM, Kim E, et al. Prepandemic physical activity and risk of COVID-19 diagnosis and hospitalization in older adults. *JAMA Netw Open*. 2024;7(2):e2355808. doi:10.1001/jamanetworkopen.2023.55808

eTable 1. Characteristics of Preventive Medicine Trial Cohort Participants and Reported COVID-19 Cases to Date
eTable 2. Adjusted Odds of COVID-19 by Level of Physical Activity Before the COVID-19 Pandemic, Restricting Follow-Up Through December 2020
eTable 3. Adjusted Odds of COVID-19 Hospitalization by Level of Physical Activity Before the COVID-19 Pandemic, Restricting Follow-Up Through December 2020
eTable 4. Adjusted Odds of COVID-19 by Level of Physical Activity Before the COVID-19 Pandemic, With Additional Adjustment for SARS-CoV-2 Vaccination Status
eTable 5. Adjusted Odds of COVID-19 Hospitalization by Level of Physical Activity Before the COVID-19 Pandemic, With Additional Adjustment for SARS-CoV-2 Vaccination Status
eTable 6. Adjusted Odds of COVID-19 by Level of Physical Activity Before the COVID-19 Pandemic, Combining Consistently Inactive and Insufficiently Active Participants in 1 Group vs Sufficiently Active
eTable 7. Adjusted Odds of COVID-19 Hospitalization by Level of Physical Activity Before the COVID-19 Pandemic, Combining Consistently Inactive and Insufficiently Active Participants in 1 Group vs Sufficiently Active
eFigure. Data Collection and Completeness of COVID-19 Outcomes

This supplementary material has been provided by the authors to give readers additional information about their work

© 2024 Muñoz-Vergara D et al. *JAMA Network Open*.

eTable 1. Characteristics of Preventive Medicine Trial Cohort Participants and Reported COVID-19 Cases to Date

	Active Trial Cohort as of 12/31/2019				Reported COVID-19 cases to date				COVID-19 hospitalized cases to date			
**Characteristic**	**Total**	**COSMOS**	**VITAL**	**WHS**	**Total**	**COSMOS**	**VITAL**	**WHS**	**Total**	**COSMOS**	**VITAL**	**WHS**
**Number**	61,557	19,655	20,151	21,751	5,890	2,020*	2,502*	1,368*	626	195	249	182
**Female, N (%)**	43,510 (71)	11,594 (59)	10,165 (50)	21,751 (100)	3,740 (64)	1,125 (56)	1,247 (50)	1,368(100)	429(69)	116 (59)	131 (53)	182 (100)
**Male, N (%)**	18,047 (29)	8,071 (41)	9,986 (50)	0 (0)	2,150 (36)	895 (44)	1,255 (50)	0 (0)	197 (31)	79 (41)	118 (47)	0 (0)
**Age categories, N (%)**												
<65 years	2,702 (4.3)	966 (5)	1,736 (8.6)	0 (0)	455 (8)	143 (7)	312 (12.5)	0 (0)	31 (5)	6 (3)	25 (10)	0 (0)
65-74 years	27,294 (44)	10,170 (52)	9,662 (48)	7,462 (34)	2,881 (49)	1,123 (56)	1,235 (49)	523 (38)	252 (40)	85 (44)	109 (44)	58 (32)
75-84 years	26,364 (43)	7,015 (36)	7,534 (37)	11,815 (54)	2,199 (37)	631 (31)	839 (34)	729 (53)	270 (43)	77 (39)	96 (39)	97 (53)
≥85 years	5,197 (8.4)	1,504 (8)	1,219 (6)	2,474 (11)	355 (6)	123 (6)	116 (4.6)	116 (8)	73 (12)	27 (14)	19 (8)	27 (15)
**Non-White (%)**	10%	8.8%	19.1%	3,4%	13%	9%	21%	3%	129 (21)	32 (16)	92 (37)	5 (3)
**Hispanic (%)**	2.3%	2.4%	3.7%	0.9%	3.2%	3%	4.4%	1.4%	27 (4.3)	8 (4)	13 (5)	6 (3)
**SARS-CoV-2 vaccine, N (%) †**	52,494 (85)	16,269 (83)	18,008 (89)	18,217 (84)	5,071 (86)	1,673 (83)	2,230 (89)	1168 (85)	481 (77)	141 (72)	197 (79)	143 (79)
**MET-hrs/week (mean, SD)[&]**	23 (26)	24 (25)	25 (29)	21 (24)	23 (26)	24 (27)	24 (27)	20 (22)	17 (27)	18 (32)	19 (26)	14 (21)
**MET-hrs/week (median, IQR)[&]**	16 (27)	17 (28)	17 (29)	14 (25)	15 (28)	16 (28)	15 (29)	13 (25)	8 (20)	8 (18)	10 (23)	6 (16)

* COVID-19 cases include those reported in REDCap surveys and in study follow-up questionnaires which reported cases through May 10, 2022.
† Information collected for at least one dose for COSMOS (March 1, 2022), VITAL (January 10, 2022), and WHS (June 1, 2021). Unknown data from 5,510 (9) participants.
& Total MET-hrs from exercise + stairs/wk as of 12/31/2019.

© 2024 Muñoz-Vergara D et al. *JAMA Network Open*.

eTable 2. Adjusted odds of COVID-19 by level of physical activity before the COVID-19 pandemic, restricting follow-up through December 2020

Physical activity category	N	Infection events	Model 1 OR (95% CI)*	Model 2 OR (95% CI)#	Model 3 OR (95% CI)†
Consistently inactive (0 – ≤ 3.5 MET-hr/wk)	12,405	584	1.00 (ref)	1.00 (ref)	1.00 (ref)
Insufficiently activity (>3.5 – < 7.5 MET-hr/wk)	6,993	327	1.03 (0.90 – 1.19)	1.02 (0.88 – 1.17)	1.01 (0.88 – 1.17)
Sufficiently active (≥ 7.5 MET-hr/wk)	42,159	1,828	0.99 (0.90 – 1.10)	0.99 (0.89 – 1.09)	0.98 (0.88 – 1.09)

* Model 1: Adjusted for demographic characteristics (i.e., sex, age, race/ethnicity, education, and income) and BMI.
# Model 2: Adjusted for demographic characteristics and lifestyle factors (i.e., BMI, smoking status and alcohol intake).
† Model 3: Fully adjusted for demographic characteristics, lifestyle factors (i.e., BMI, smoking status and alcohol intake), and comorbidities/medication use (i.e., history of diabetes, hypertension, malignant cancer, myocardial infarction, or stroke and use of NSAIDs, aspirin, and statins medication).

© 2024 Muñoz-Vergara D et al. *JAMA Network Open*.

eTable 3. Adjusted odds of COVID-19 hospitalization by level of physical activity before the COVID-19 pandemic, restricting follow-up through December 2020

Physical activity category	N	Severe events	Model 1 OR (95% CI)*	Model 2 OR (95% CI)#	Model 3 OR (95% CI)†
Consistently inactive (0 – ≤ 3.5 MET-hr/wk)	12,405	118	1.00 (ref)	1.00 (ref)	1.00 (ref)
Insufficiently activity (>3.5 – < 7.5 MET-hr/wk)	6,993	46	0.82 (0.58 – 1.17)	0.83 (0.58 – 1.19)	0.87 (0.61 –1.24)
Sufficiently active (≥ 7.5 MET-hr/wk)	42,159	167	0.61 (0.47 – 0.79)	0.63 (0.49 – 0.82)	0.64 (0.49 – 0.83)

* Model 1: Adjusted for demographic characteristics (i.e., sex, age, race/ethnicity, education, and income) and BMI.
# Model 2: Adjusted for demographic characteristics and lifestyle factors (i.e., BMI, smoking status and alcohol intake).
† Model 3: Fully adjusted for demographic characteristics, lifestyle factors (i.e., BMI, smoking status and alcohol intake), and comorbidities/medication use (i.e., history of diabetes, hypertension, malignant cancer, myocardial infarction, or stroke and use of NSAIDs, aspirin, and statins medication).

© 2024 Muñoz-Vergara D et al. *JAMA Network Open*.

eTable 4. Adjusted odds of COVID-19 by level of physical activity before the COVID-19 pandemic, with SARS-CoV-2 vaccination status added to the model

Physical activity category	N	Infection events	Model 3 OR (95% CI)†
Consistently inactive (0 – ≤ 3.5 MET-hr/wk)	12,405	1,293	1.00 (ref)
Insufficiently activity (>3.5 – < 7.5 MET-hr/wk)	6,993	699	0.95 (0.85 – 1.05)
Sufficiently active (≥ 7.5 MET-hr/wk)	42,159	3,898	0.89 (0.82 – 0.96)

† Fully adjusted for demographic characteristics, lifestyle factors (i.e., BMI, smoking status and alcohol intake), and comorbidities/medication use (i.e., history of diabetes, hypertension, malignant cancer, myocardial infarction, or stroke and use of NSAIDs, aspirin, and statins medication) (Model 3), as well as SARS-CoV-2 vaccination status.

© 2024 Muñoz-Vergara D et al. *JAMA Network Open*.

eTable 5. Adjusted odds of COVID-19 hospitalization by level of physical activity before the COVID-19 pandemic, with SARS-CoV-2 vaccination status added to the model

Physical activity category	N	Hospitalization events	Model 3 OR (95% CI)†
Consistently inactive (0 – ≤ 3.5 MET-hr/wk)	12,405	203	1.00 (ref)
Insufficiently activity (>3.5 – < 7.5 MET-hr/wk)	6,993	91	0.96 (0.73 – 1.26)
Sufficiently active (≥ 7.5 MET-hr/wk)	42,159	332	0.74 (0.60 – 0.92)

† Fully adjusted for demographic characteristics, lifestyle factors (i.e., BMI, smoking status and alcohol intake), and comorbidities/medication use (i.e., history of diabetes, hypertension, malignant cancer, myocardial infarction, or stroke and use of NSAIDs, aspirin, and statins medication) (Model 3), as well as SARS-CoV-2 vaccination status.

© 2024 Muñoz-Vergara D et al. *JAMA Network Open*.

eTable 6. Adjusted odds of COVID-19 by level of physical activity before the COVID-19 pandemic, combining consistently inactive and insufficiently active participants in one group vs. sufficiently active

Physical activity category	N	Infection events	Model 3 OR (95% CI)†
Insufficiently activity (0 – < 7.5 MET-hr/wk)	19,398	1,992	1.00 (ref)
Sufficiently active (≥ 7.5 MET-hr/wk)	42,159	3,898	0.92 (0.86 – 0.98)

† Fully adjusted for demographic characteristics, lifestyle factors (i.e., BMI, smoking status and alcohol intake), and comorbidities/medication use (i.e., history of diabetes, hypertension, malignant cancer, myocardial infarction, or stroke and use of NSAIDs, aspirin, and statins medication) (Model 3).

© 2024 Muñoz-Vergara D et al. *JAMA Network Open*.

eTable 7. Adjusted odds of COVID-19 hospitalization by level of physical activity before the COVID-19 pandemic, combining consistently inactive and insufficiently active participants in one group vs. sufficiently active

Physical activity category	N	Hospitalization events	Model 3 OR (95% CI)†
Insufficiently activity (0 – < 7.5 MET-hr/wk)	19,398	294	1.00 (ref)
Sufficiently active (≥ 7.5 MET-hr/wk)	42,159	332	0.74 (0.62 – 0.89)

† Fully adjusted for demographic characteristics, lifestyle factors (i.e., BMI, smoking status and alcohol intake), and comorbidities/medication use (i.e., history of diabetes, hypertension, malignant cancer, myocardial infarction, or stroke and use of NSAIDs, aspirin, and statins medication) (Model 3).

© 2024 Muñoz-Vergara D et al. *JAMA Network Open*.

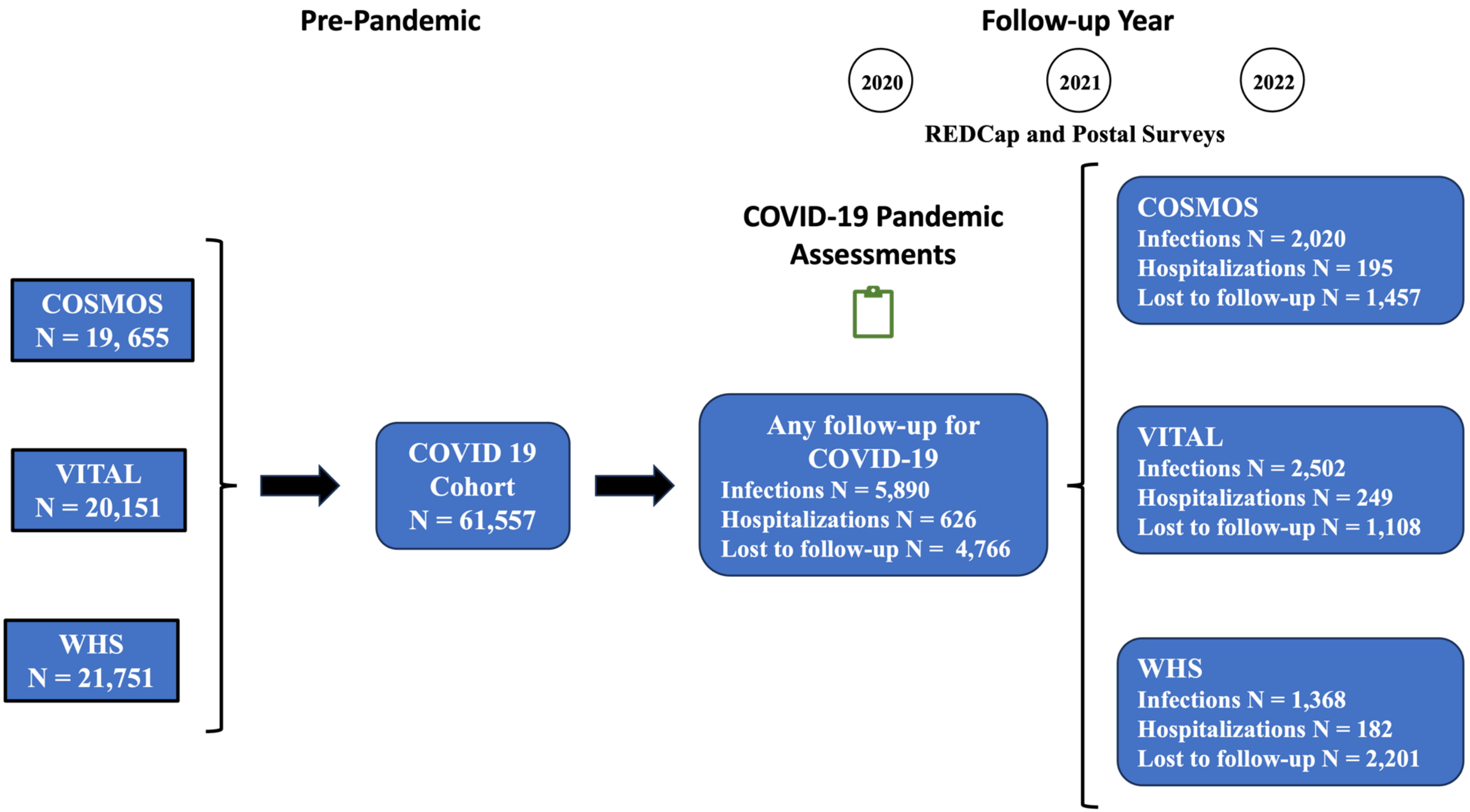

eFigure. Data collection and completeness of COVID-19 outcomes

© 2024 Muñoz-Vergara D et al. *JAMA Network Open*.
