# Supplementary material for: Prepandemic Physical Activity and Risk of COVID-19 Diagnosis and Hospitalization in Older Adults
Source: JAMA Netw Open. 2024 Feb 13;7(2):e2355808. doi: 10.1001/jamanetworkopen.2023.55808 (PMC10865155; doi:10.1001/jamanetworkopen.2023.55808)
Supplement: Supplement 2. — Data Sharing Statement [file jamanetwopen-e2355808-s002.pdf]

Data Sharing Statement

Muñoz-Vergara. Prepandemic Physical Activity and Risk of COVID-19 Diagnosis and Hospitalization in Older Adults. *JAMA Netw Open*. Published February 13, 2024. doi:10.1001/jamanetworkopen.2023.55808

Data
Data available: No
